# Transcriptomic evidence that von Economo neurons are regionally specialized extratelencephalic-projecting excitatory neurons

**DOI:** 10.1038/s41467-020-14952-3

**Published:** 2020-03-03

**Authors:** Rebecca D. Hodge, Jeremy A. Miller, Mark Novotny, Brian E. Kalmbach, Jonathan T. Ting, Trygve E. Bakken, Brian D. Aevermann, Eliza R. Barkan, Madeline L. Berkowitz-Cerasano, Charles Cobbs, Francisco Diez-Fuertes, Song-Lin Ding, Jamison McCorrison, Nicholas J. Schork, Soraya I. Shehata, Kimberly A. Smith, Susan M. Sunkin, Danny N. Tran, Pratap Venepally, Anna Marie Yanny, Frank J. Steemers, John W. Phillips, Amy Bernard, Christof Koch, Roger S. Lasken, Richard H. Scheuermann, Ed S. Lein

**Affiliations:** 1 Allen Institute for Brain Science, Seattle, WA USA; 2 J. Craig Venter Institute, La Jolla, CA USA; 3 Department of Physiology and Biophysics, University of Washington, Seattle, WA USA; 4 Department of Neurological Surgery, University of Washington, Seattle, WA USA; 5 The Ben and Catherine Ivy Center for Advanced Brain Tumor Treatment, Swedish Neuroscience Institute, Seattle, WA USA; 6 J. Craig Venter Institute, Rockville, MD USA; 7 Illumina, Inc., San Diego, CA USA; 8 Department of Pathology, University of California, San Diego, CA USA

**Keywords:** Cellular neuroscience, Genetics of the nervous system, Molecular neuroscience

## Abstract

von Economo neurons (VENs) are bipolar, spindle-shaped neurons restricted to layer 5 of human frontoinsula and anterior cingulate cortex that appear to be selectively vulnerable to neuropsychiatric and neurodegenerative diseases, although little is known about other VEN cellular phenotypes. Single nucleus RNA-sequencing of frontoinsula layer 5 identifies a transcriptomically-defined cell cluster that contained VENs, but also fork cells and a subset of pyramidal neurons. Cross-species alignment of this cell cluster with a well-annotated mouse classification shows strong homology to extratelencephalic (ET) excitatory neurons that project to subcerebral targets. This cluster also shows strong homology to a putative ET cluster in human temporal cortex, but with a strikingly specific regional signature. Together these results suggest that VENs are a regionally distinctive type of ET neuron. Additionally, we describe the first patch clamp recordings of VENs from neurosurgically-resected tissue that show distinctive intrinsic membrane properties relative to neighboring pyramidal neurons.

## Abstract (web summary)

Little is known about von Economo neurons, which have been described in a subset of mammals and appear to be selectively lost in several human neurological diseases. Here, authors reveal the gene expression profile of these cells and show that they are likely long-distance projection neurons.

## Introduction

von Economo neurons (VENs) are a morphologically-defined neuron type with a characteristic large, spindle-shaped cell body. They possess thick bipolar dendrites with limited branching and a moderate density of spines, and often have an axon initial segment that emanates from the side of the cell body^1–3^. VENs have been described in several large-brained mammals, such as humans, great apes, macaques, cetaceans, cows, and elephants, but not in rodents^1,4–11^. In humans, they are restricted to the anterior cingulate (ACC), frontoinsular (FI), and medial frontopolar regions of cerebral cortex^12^, while in some other species they are also found in the frontal and occipital poles^13^ and may not be restricted to layer 5. Fork cells, another distinctive morphologically-defined neuron type, are often found in the same brain regions as VENs and are similarly characterized by a single large basal dendrite, but differ from VENs by having a divided apical dendrite^1,14^. VENs and fork cells appear to be selectively vulnerable neuron types, as loss of these cells has been observed in behavioral variant frontotemporal dementia (bvFTD)^15–17^. Loss of VENs has also been observed in several neuropsychiatric disorders, including schizophrenia^18^ and suicidal psychosis^19^, as well as in autism^20^, agenesis of the corpus callosum^21^, and possibly Alzheimer’s disease^22,23^.

Very little is known about VEN cellular phenotypes beyond their hallmark morphology, especially in human cortex. Human FI and ACC neurosurgical resections are extremely rare for functional studies, and VEN sparsity without some form of genetically-based labeling makes their analysis difficult. Molecular analyses of human VENs have been more fruitful since these techniques can be applied to postmortem human tissues. For example, a recent study using in situ hybridization (ISH) data from the Allen Human Brain Atlas identified *ADRA1A*, *GABRQ*, and *VMAT2* as VEN marker genes^24^, and a study using laser microdissection of VENs followed by RNA-sequencing identified additional potential VEN marker genes^25^. VENs have also been reported to express serotonin receptor 2B (*HTR2B*)^26^, dopamine receptor D3 (*DRD3*)^26^, and the Schizophrenia-associated protein DISC1^4,27^. Additionally, they express transcription factors *FEZF2* and *CTIP2*^28^, which are required for generating subcortical projection neurons in mice^29^, and this has been used as evidence that VENs are subcortically-projecting neurons. However, *Fezf2* is not specific for ET neurons but is also expressed in near-projecting pyramidal neurons in adult mouse^30^, and expression of many cellular marker genes is not conserved between mouse and human^31,32^. Here we refer to subcortically-projecting neurons as extratelencephalic-projecting excitatory neurons (ET)^33^, which are also sometimes referred to as pyramidal tract neurons and subcerebral projection neurons^34,35^. Importantly, we acknowledge that ET neurons may not strictly project to subcortical structures and may have telencephalic collaterals. In rhesus monkey, tract-tracing studies suggest that VENs might project to ipsilateral ACC and contralateral anterior insula^4,36^, as well as to more distant subcortical targets in the pons and midbrain^27,28^. Furthermore, many of the reported markers of VENs are not exclusive to these cells but are also expressed in fork cells and pyramidal-shaped neurons. This highly incomplete characterization leaves unresolved many questions about whether morphologically-defined VENs represent a molecularly-distinct cell type and what their other properties are.

Single cell RNA-sequencing (scRNA-seq) has emerged as an effective strategy for classifying and characterizing cell types in complex brain tissues, and single nucleus (sn) RNA-seq can be used on frozen postmortem human brain specimens^37,38^. Applied to cortex, this approach reveals a high degree of cellular diversity, with upwards of 100 transcriptomically-defined cell types in any cortical area^30,32,39,40^. Furthermore, these data enable quantitative alignment of cell types across brain regions and between species to predict identity by transcriptional similarity using new computational strategies for mapping of transcriptomic types between datasets^41–43^. Such alignment enables prediction of cellular properties and projection targets in human based on properties described in well-studied mouse cell types^32^.

To reveal the transcriptomic signature and predict properties of VENs, we performed snRNA-seq on nuclei from layer 5 of FI and compared to similar data from human temporal cortex and two cortical areas in mouse. We find a single transcriptomic cluster expressing several known markers for VENs that aligns with ET neurons in mouse cortex, as well as a putative transcriptomically-defined ET cluster in human temporal cortex that has a distinctive regional signature compared to FI. We identify many novel markers for this cluster and demonstrate that they are co-expressed in a combination of pyramidal neurons, VENs, and fork cells. Finally, we present a case study with the first electrophysiological recordings of putative VENs, and show that they have distinctive intrinsic membrane properties from neighboring layer 5 pyramidal neurons.

## Results

### Transcriptomic cell types in layer 5 of FI

**Fig. 1:**
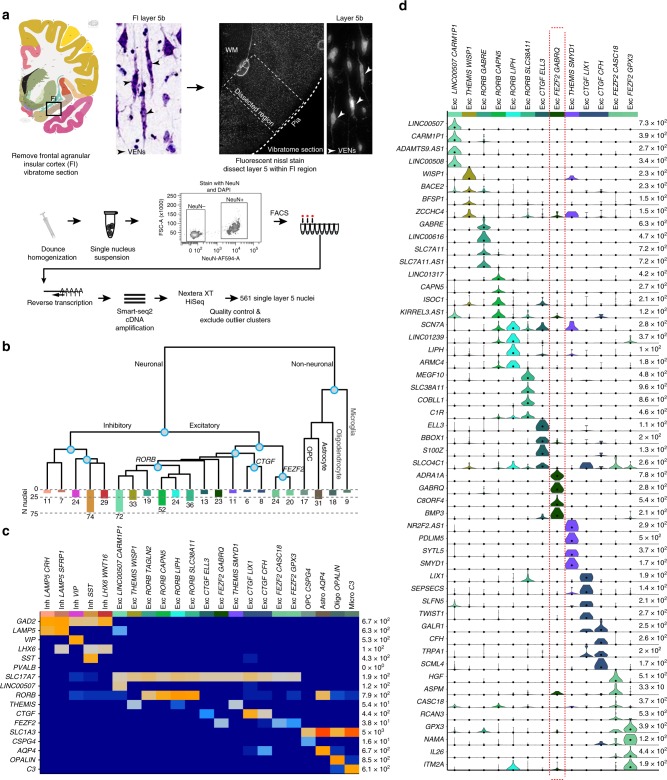
Cell type characterization in human frontal agranular insular cortex (FI). **a** Schematic diagram illustrating nuclei isolation from postmortem human brain specimens. The FI region was isolated, vibratome sectioned, stained with fluorescent Nissl, and layer 5 was dissected and processed for nuclei isolation, fluorescence-activated cell sorting (FACS), and RNA-sequencing. Examples of cells with morphologies typical of von Economo neurons (VENs) are shown in the images of Nissl-stained tissues (arrowheads). Human brain image © 2010 Allen Institute for Brain Science. Allen Human Brain Atlas. Available from: http://human.brain-map.org/. In total 561 single layer 5 neurons passed quality control. **b** Hierarchical representation of 18 neuronal (5 inhibitory, 13 excitatory) and 4 non-neuronal transcriptomic cell types based on median cluster expression. Major cell classes are labeled at branch points in the dendrogram. The bar plot and associated numbers below the dendrogram represent the number of nuclei within each cluster. Cluster-specific colors and labels are used in all subsequent figures. **c** Heatmap showing the expression of cell class marker genes across all clusters. Maximum expression values for each gene are listed on the far right-hand side of the plot. Gene expression values are quantified as counts per million of intronic plus exonic reads and displayed on a log10 scale, using a blue-white-red color scheme with blue = 0 and red = the maximum value in the plot (5 × 10^3^). **d** Violin plots showing expression of four marker genes per excitatory cluster. Each row represents a gene, black dots show median gene expression within clusters, and maximum expression values for each gene are shown on the right-hand side of each row. Gene expression values are displayed on a linear scale. Box indicates putative VEN cluster.

We employed snRNA-seq^37,38^ to profile nuclei from FI of two postmortem human brain specimens (Fig. 1a) as previously described^32,44^. Briefly, layer 5 was microdissected from fluorescent Nissl-stained vibratome sections of FI and nuclei were liberated from tissue by Dounce homogenization. NeuN staining and fluorescence-activated cell sorting (FACS) were used to enrich for neuronal (NeuN+) and non-neuronal (NeuN−) nuclei (Supplementary Fig. 1a). RNA-sequencing was carried out using Smart-seq2, Nextera XT, and HiSeq sequencing. In total 879 nuclei that passed initial quality control metrics were processed for snRNA-seq. These nuclei were sequenced to a median of 4 million mapped reads per nucleus. Median gene detection (expression >0) was 10,339 genes per nucleus for excitatory neurons, 9,426 for inhibitory neurons, and 6,146 for non-neuronal cells, consistent with previous reports^30,32,44^ (Supplementary Fig. 1b).

Iterative clustering was performed as described in refs. ^30,32,44^) to group nuclei by gene expression similarity. Briefly, high variance genes were identified while accounting for gene dropouts, expression dimensionality was reduced with principal components analysis (PCA), and nuclei were clustered using Jaccard-Louvain community detection. Clusters containing cells from only a single donor as well as nuclei mapping to low-quality outlier clusters (*n* = 318 nuclei; approximately half for each filter) were excluded from further analysis, leaving a total of 561 high quality nuclei. We identified a robust set of 22 transcriptomically-defined clusters (Fig. 1b) that contained cells from both donors at roughly comparable proportions within broad classes (Supplementary Fig. 1c, d). Clusters were named by combining the most highly-expressed broad class marker with the most highly specific marker for that cluster^32^. Five inhibitory neuron types spanning all expected subclasses (two *LAMP5* types, *VIP*, SST, and a *LHX6*+/*SST*− cluster corresponding to *PVALB* interneurons), 13 excitatory neuron types, and four major non-neuronal cell types (oligodendrocyte precursor cells, oligodendrocytes, astrocytes, and microglia) were identified (Fig. 1c). Given the small sample size in our study, we predict that deeper sampling inclusive of all layers in FI would reveal more refined cell type resolution, consistent with previous studies that deeply sampled mouse^30^ and human cortex^32^.

**Fig. 2:**
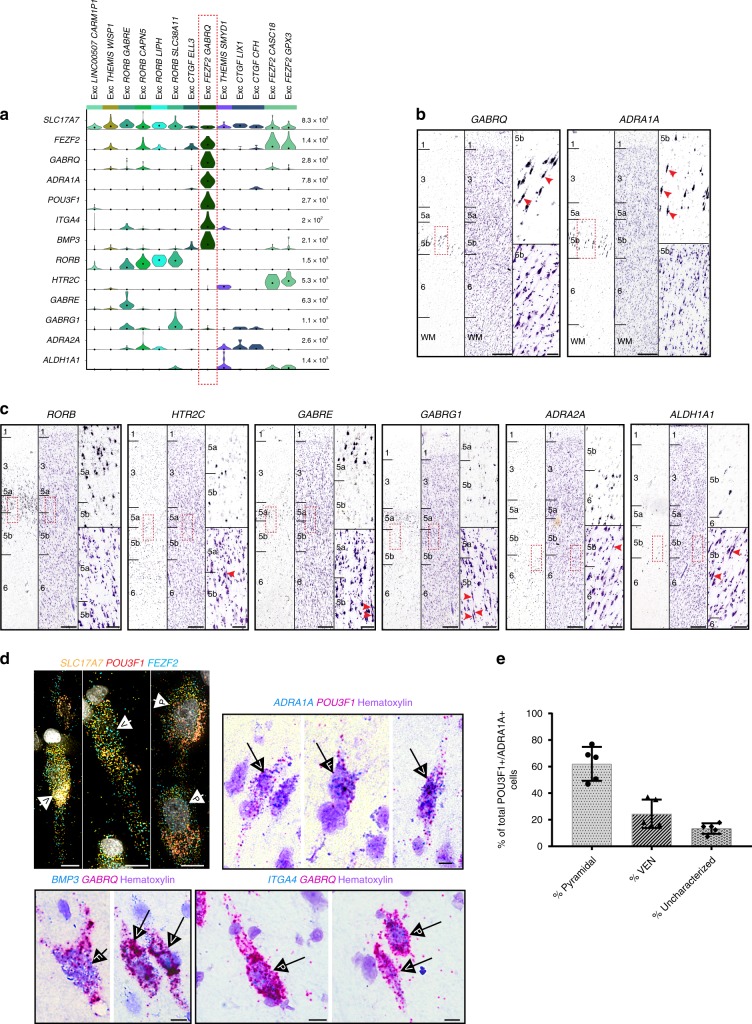
Identifying a transcriptomic cell type that corresponds to von Economo neurons (VENs) in situ. **a** Violin plots showing distributions of genes further examined by in situ hybridization (ISH). Each row represents a gene, black dots indicate median gene expression within clusters, and maximum gene expression values are shown on the right-hand side of each row. Gene expression values are displayed on a linear scale. The EXC *GABRQ FEZF2* type expresses *GABRQ* and *ADRA1A*, previously defined markers of VENs. **b** Chromogenic single gene ISH from the Allen Human Brain Atlas (http://human.brain-map.org/) for *GABRQ* and *ADRA1A* confirms a subset of layer 5b cells expressing these genes have spindle-shaped cell bodies typical of VENs (red arrows). The nearest Nissl-stained section is shown for each ISH image for laminar context. **c** ISH from the Allen Human Brain Atlas (http://human.brain-map.org/) for genes expressed in other excitatory neuron types revealed by our analyses. Genes are expressed in and around layer 5 of FI but labeled cells lack spindle-shaped cell bodies typical of VENs. Red arrows in the nearest Nissl-stained section for each ISH image show representative examples of cells with VEN morphology in the approximate region highlighted in the neighboring ISH image (red rectangle). Scale bars in **b**, **c** low magnification, 150 μm, high magnification 50 μm. **d** Multiplex fluorescent ISH (top left) and double chromogenic ISH for marker genes of Exc *FEZF2 GABRQ*. Cells with pyramidal (P), VEN (V), and fork (F) morphologies are indicated by labeled arrows in each image. Scale bars, 10 μm. **e** Quantification of the proportion of *ADRA1A*+*, POU3F1*+ cells with pyramidal vs. VEN morphologies (*n* = 5 human donors). Cells lacking defining features of these morphological classes were called uncharacterized. Bars show the mean and error bars the standard deviation. Individual data points are overlaid on each bar plot.

Excitatory clusters in FI expressed broad class markers previously identified in human middle temporal gyrus (MTG) (Fig. 1c, d)^32^. One cluster had high expression of the MTG upper layer marker *LINC00507* and likely represents deep layer 3 pyramidal neurons sampled at the layer 3/5a boundary, since FI is agranular and does not contain layer 4. Three clusters expressed *CTGF*, a canonical marker for deep layer 6 neurons in mouse that has more widespread expression in human layer 6^31^, suggesting these clusters represent cells captured at the layer 5b/6 border. Two clusters highly expressed *THEMIS*, which is also expressed in layer 5 and layer 6 excitatory neuron types in MTG^32^. Four clusters expressed *RORB*, which marks a subset of cells localized throughout layers 3–5 in MTG^32^ and has a similar pattern of expression in FI (Fig. 2). Finally, we found 3 clusters with high expression of *FEZF2*, previously shown to be expressed in VENs^28^, subcortically-projecting and near-projecting excitatory neurons in mouse cortex^30^, and several deep layer excitatory types in human MTG^32^.

### Identifying a transcriptomic cell type corresponding to VENs

To characterize each transcriptomic type and determine whether one might represent VENs, we examined selective marker genes for each excitatory cluster (the top four markers per cluster are shown in Fig. 1d). One cluster, Exc *FEZF2 GABRQ*, specifically expressed the reported VEN and fork cell markers *GABRQ* and *ADRA1A*^24^, suggesting that this cluster, but not the other two *FEZF2*+ clusters (Fig. 1c), likely included VENs. Exc *FEZF2 GABRQ* also had the largest number of expressed genes (Supplementary Fig. 1b), suggesting high RNA content and perhaps correlated with the reported large size of VENs^1,12^. To confirm that Exc *FEZF2 GABRQ* included VENs, we looked for genes selective for one or more excitatory cell types in our dataset that also had existing ISH data in the Allen Human Brain Atlas (http://human.brain-map.org/)^31,45^ (Fig. 2a). As previously reported^24^ and supporting the identification of Exc *FEZF2 GABRQ* as the cluster containing VENs, ISH for *GABRQ* and *ADRA1A* showed that a subset of cells in layer 5b expressing these genes have spindle-shaped cell bodies typical of VENs (Fig. 2b). However, not all cells labeled with *GABRQ* and *ADRA1A* had spindle-shaped cell bodies, indicating that these genes do not exclusively mark VENs. In contrast, ISH for genes expressed in other excitatory neuron types did not label cells with obviously spindle-shaped cell bodies even though Nissl-stained sections nearest to those used for ISH confirmed that cells with VEN morphology were present in the region examined with ISH (Fig. 2c; a subset of VENs in the Nissl images are labeled with an arrow for reference). In particular, both *HTR2C* and *ALDH1A1*, which were expressed in the other two *FEZF2*+ cell types that we found, did not label any spindle-shaped cells.

To further validate and explore the morphological cell types comprising the Exc *FEZF2 GABRQ* cluster, we performed multiplex fluorescent (mFISH) and double chromogenic (dISH) ISH for cluster-specific marker genes (Fig. 2a, d). Consistent with single gene ISH for *GABRQ* and *ADRA1A*, we find that pyramidal-shaped neurons, fork cells, and VENs are all labeled with combinations of specific marker genes for Exc *FEZF2 GABRQ* suggesting that this single transcriptomic type contains a mixture of morphological cell types (Fig. 2d). To quantify the proportions of cells in the Exc *FEZF2 GABRQ* type that have these different morphologies, we performed dISH staining for *ADRA1A* and the cluster-specific marker *POU3F1* because high expression of this gene in combination with significant amplification of signal intensity in the dISH method both distinctly labels the Exc *FEZF2 GABRQ* type and highlights the morphology of labeled cells (Fig. 2d). Double-positive cells were classed as pyramidal, VEN, or uncharacterized (cells that lacked defining morphological features and were likely bisected by the plane of section, see the “Methods” section) in FI tissues from 5 different human donors. Fork cells were extremely rare and were not explicitly quantified. Our results show that of all *ADRA1A* and *POU3F1* double-positive cells, ~60% had pyramidal morphology compared with ~25% that had VEN morphology, confirming that morphologically-defined VENs represent only a subset of the neurons that comprise the Exc *FEZF2 GABRQ* type. To the best of our knowledge, *POU3F1*, *BMP3*, and *ITGA4* represent novel, validated markers for VENs (Fig. 2d).

We next attempted to further divide the 23 nuclei assigned to the Exc *FEZF2 GABRQ* cluster into subgroups that potentially could reflect cells with different morphological identities. Reclustering our data with more permissive parameter settings yielded additional sub-clustering of other excitatory cell types, but no change in the Exc *FEZF2 GABRQ* cluster (Supplementary Fig. 2a). Similarly, a supervised analysis using genes previously reported as differentially expressed between VEN and pyramidal-shaped neurons^46^ did not reveal greater heterogeneity in these nuclei than was found using other gene sets (Supplementary Fig. 2b, c). Based on these analyses, there do not appear to be any obvious gene expression differences in the morphologically-distinct cells that comprise the Exc *FEZF2 GABRQ* cluster; however, due to the small number of cells identified in this cluster (*N* = 23), we cannot rule out subtle transcriptional differences that could divide this cluster into multiple subtypes with additional sampling.

### VENs predicted to be ET-projecting excitatory neurons

New methods that enable alignment of cells between data sets based on gene expression profiles can be used to align cell types across cortical regions and across species^41–43^. This provides a mechanism for predicting cellular properties of human cell types based on measurements made in homologous cell types from model systems. For example, performing retrograde labeling and scRNA-seq on the same cells (i.e. Retro-seq) enables determination of the long-range projection specificity of excitatory cell types in mouse cortex^30^. Previously, we showed that nearly all transcriptomically-defined cell classes and subclasses identified in human MTG can be aligned with transcriptomically-defined types in mouse anterior lateral motor cortex (ALM) and primary visual cortex (VISp), even if other features are distinct between species^32^.

**Fig. 3:**
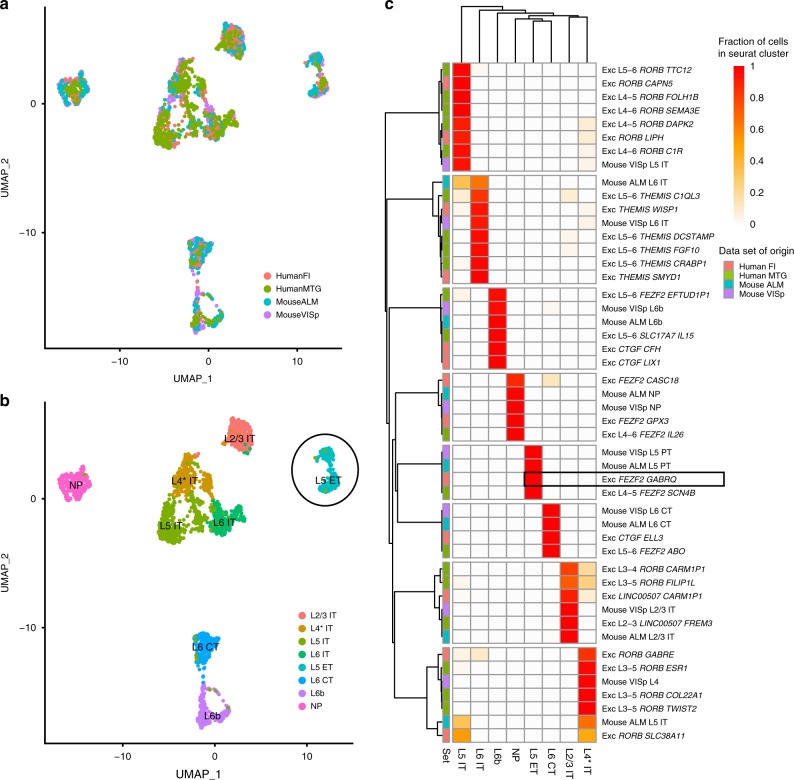
Evolutionary conservation of cell types between human and mouse predicts that VENs project sub-cortically. **a**, **b** Excitatory neurons in human FI (red), human MTG (green), mouse ALM (cyan), and mouse VISp (purple) were integrated and aligned using Seurat v3^41^ with default parameters, and visualized using UMAP. **a** Cells from each data-set co-cluster, indicating good matching of types between brain regions and species. **b** Eight Seurat clusters were identified using the Louvain algorithm and labeled based on expected cortical layer and projection target (as described in **c**). **c** Membership of cells from excitatory clusters in each data set in the Seurat clusters. Colors indicate the fraction of total cells per cluster assigned to each Seurat cluster (rows sum to 1). Data set clusters are grouped based on maximal fraction of cells in the cluster. Cortical layer of each cluster are inferred based on predominant cortical layer of cells from mouse and human data sets, with the exception of the layer 4 cluster (L4* IT) which primarily includes cells from layer 5 in structures without a layer 4. Projection targets of clusters are inferred based on known projection targets of clusters in mouse ALM and VISp (IT—intratelencephalic, ET—extratelencephalic, NP—near-projecting, CT—corticothalamic). The box highlights that Exc *FEZF2 GABRQ* is part of the L5 ET cluster.

To shed light on the cellular properties of the Exc *FEZF2 GABRQ* type, we combined the present data from human FI with representative sets of cells from human MTG^32^ and mouse ALM and VISp^30^ into an integrated reference using Seurat (V3.0)^41,42^. Only excitatory cells from each data set were included in the assembly, and cells from mouse data sets were grouped based on subclass, which combines cell types with the same predominant layer of soma location and long-range projection targets^30^. Eight clusters were identified using Seurat, which each contained cells from all four data sets (Fig. 3a) and that matched with the groupings visualized through UMAP dimensionality reduction (Fig. 3b). More importantly, nearly all cells from mouse were mapped to the cluster in the joint assembly that matched their initially assigned subclass (Fig. 3c), with one exception. As reported in mouse ALM^30^, we identified one cluster in agranular human FI (Exc *RORB SLC38A11*) whose best match was intratelencephalic (IT) layer 4 clusters in human MTG and mouse VISp. Furthermore, for almost all MTG clusters the subclass assignments here match those previously reported using different alignment methodologies (compare Fig. 3c with Fig. 5 from ref. ^32^).

We find that Exc *FEZF2 GABRQ* co-clusters with Exc L4-5 *FEZF2 SCN4B* from human MTG and with all layer 5 ET clusters from mouse VISp and ALM (Fig. 3c), which we confirmed by running Seurat with different parameter settings and through an independent correlation-based mapping strategy (see the “Methods” section). This result suggests that VENs are part of a cluster of neurons that are predicted to have deep subcortical projections, though further experiments would be necessary to confirm this hypothesis. Interestingly, the other two *FEZF2+* clusters in FI co-cluster with near-projecting neurons in mouse, suggesting that they may not have long-range projections despite the developmental role of *FEZF2* in specifying subcortical projection neurons^29^. A summary of these results, including common markers between species, is shown in Supplementary Fig. 3.

### Molecular features of putative ET neurons

**Fig. 4:**
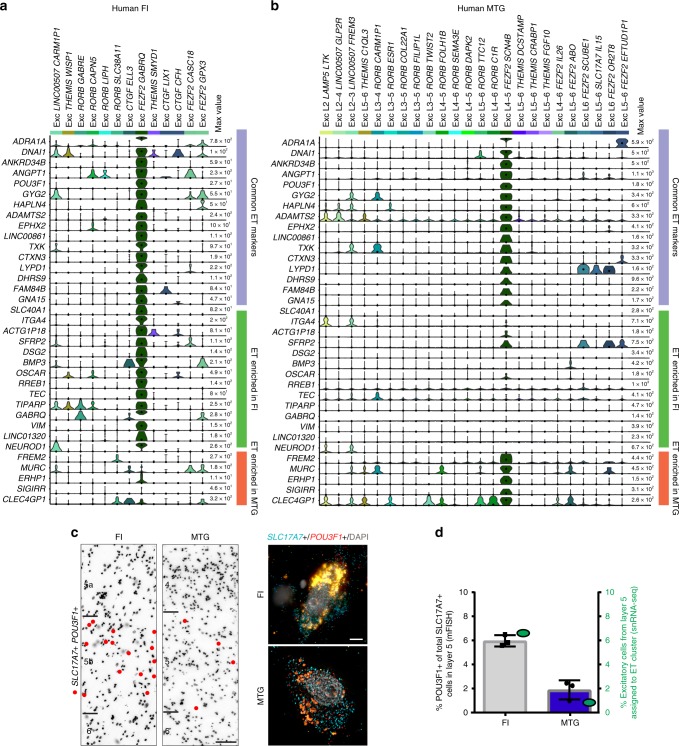
Putative extratelencephalic (ET) cells in human frontoinsula (FI) and middle temporal gyrus (MTG) share many common markers but differ in frequency. **a**, **b** Violin plots in human FI (**a**) and MTG (**b**) showing expression of all genes enriched in Exc *FEZF2 GABRQ*, and a subset of genes enriched in the corresponding cluster in human MTG that show divergent patterning between these regions (see the “Methods” section), including many novel marker genes. Gene expression values are displayed on a log2 scale. **c** Representative inverted images of DAPI-stained sections of FI and MTG. Red dots depict the locations of cells labeled using multiplex fluorescent in situ hybridization (mFISH) for putative ET marker gene *POU3F1* and *SLC17A7*. Scale bars in **c**: DAPI images 50 μm, mFISH images 10 μm. **d** Black, quantification of the proportion of *SLC17A7*+ cells expressing the ET marker *POU3F1* in FI and MTG expressed as a fraction of the total number of excitatory (*SLC17A7*+) cells in layer 5 of either region. Individual data points are represented by black squares (FI) or black dots (MTG). Green, comparable quantification of the fraction of excitatory neurons dissected from layer 5 that are assigned to the ET clusters Exc L4-5 *FEZF2 SCN4B* (MTG) or Exc *FEZF2 GABRQ* (FI). By both mFISH and single nucleus RNA-seq (snRNA-seq), a higher fraction of putative ET cells is found in FI than MTG. Bars show the mean and error bars the standard deviation.

While several VEN marker genes have been previously described^24,28,46^, we find that, although most of these genes are expressed in Exc *FEZF2 GABRQ*, very few are specific to this cluster but rather are expressed in several or many other excitatory neuron types (Supplementary Fig. 4). To describe a more refined set of genes selectively expressed in VENs and other putative ET neurons, we performed differential expression analysis comparing Exc *FEZF2 GABRQ* to all other excitatory clusters (see the “Methods” section) and identified 30 genes selectively expressed in Exc *FEZF2 GABRQ* (Fig. 4a). These genes included reported markers for VENs such as *GABRQ* and *ADRA1A*, as well as many novel markers. Several genes appear to be common ET cell markers in mouse and human, including *FAM84B*, *POU3F1*, and *ANKRD34B* (Supplementary Fig. 3), although many more show divergent patterning between species consistent with previous comparisons of mouse and human cell types^32^.

Approximately half of genes enriched in Exc *FEZF2 GABRQ* were similarly enriched in the matching putative ET cluster from human MTG (e.g., *ADRA1A*) (see the “Methods” section, Fig. 4b). However, region-specific genes were apparent for both FI (Exc *FEZF2 GABRQ*) and MTG (Exc L4-5 *FEZF2 SCN4B*), consistent with reported variation of excitatory neurons across cortical areas^30^ (Fig. 4a, b). We note that a comparable number of MTG-enriched genes as FI-enriched genes were found, but only a subset of MTG-enriched genes is shown in Fig. 4 to highlight our FI results. Interestingly, while the putative-ET cluster was highly distinct transcriptomically in each data set, it also had the highest Spearman correlation between each pair of data sets (see the “Methods” section), suggesting that the morphological specialization of VENs likely is not accompanied by transcriptomic specialization that is comparable in scope.

SnRNA-seq data suggested that the proportion of putative ET neurons may also vary between MTG and FI (Fig. 4c). To further examine this difference in situ we used mFISH to count the fraction of total excitatory cells (*SLC17A7*+) in layer 5 that also express the specific putative ET marker gene *POU3F1* (Fig. 4c, d). In agreement with snRNA-seq data, mFISH counts showed that a substantially higher fraction of putative ET cells was found in FI than in MTG. Together these results indicate that, while the primary features of putative ET neurons in human are conserved across cortical areas, ET neurons show some transcriptomic differences between cortical areas, and putative ET neurons in FI appear to be more abundant and have more diverse cellular morphologies than those in MTG.

### Intrinsic membrane properties of putative VENs

**Fig. 5:**
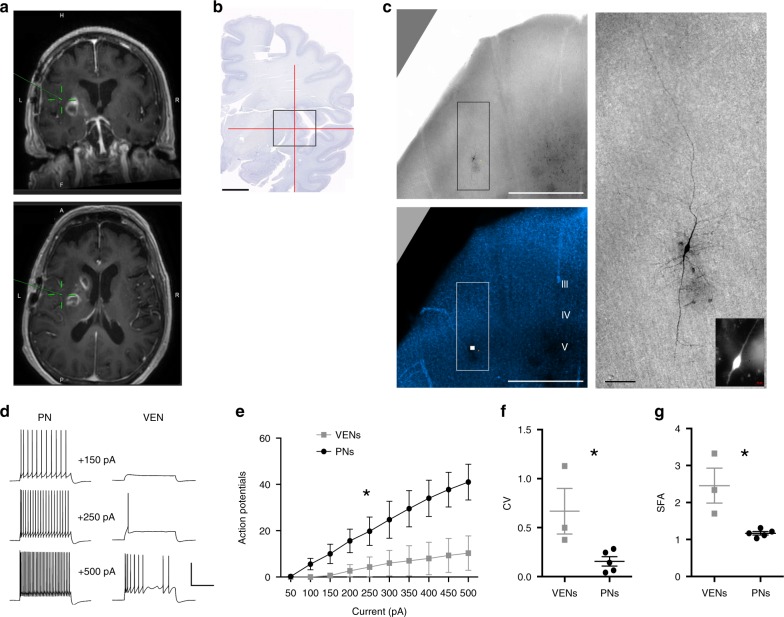
Distinctive electrophysiological properties of putative L5 VENs in ex vivo insula brain slices from a human neurosurgery patient. **a** MRI scan indicating the location of the excised insula tissue specimen for research. **b** Best matched location in the Allen 2D coronal human brain reference atlas, with crosshairs centered on the short insular gyrus. Scale bar: 1 cm. **c** Biocytin-filled putative VEN in L5 of an ex vivo insula brain slice. Low magnification brightfield and DAPI image confirms the L5 location of the neuron. The boxed region bounding the biocytin-filled neuron is expanded at right. Inset: image of Alexa dye fill following patch clamp recording in live tissue. Scale bars: 1 mm and 100 µm. **d** Example traces of action potential firing pattern in response to current injection steps for a representative pyramidal neuron (PN) and VEN. Scale bars: 50 pA, 500 msec. **e** Summary plot of action potential firing in response to current injection steps. *p < 0.0001, 2-way ANOVA. **f** Summary plot of coefficient of variation (CV) for VENs vs. PNs. *p < 0.05, Mann–Whitney. **g** Summary plot of spike frequency adaptation (SFA) for VENs vs. PNs. *p < 0.05, Mann–Whitney. Center lines show mean and error bars show standard error of the mean.

ET neurons possess distinctive intrinsic membrane properties compared to neighboring non-ET neurons^34,47^. To test whether VENs also have distinctive electrophysiological properties, we took advantage of a very rare opportunity to perform single neuron patch clamp recordings in human insula ex vivo brain slices from a single human donor. In this case study, peri-tumor insula tissue was removed from the brain of a 68-year-old female patient to access a deep brain tumor located in the left insula/putamen region (Fig. 5a, b). We performed whole-cell patch clamp recordings from large spindle-shaped neurons (putative VENs) in layer 5 (*n* = 3 cells) and nearby (presumably non-ET) pyramidal neurons for comparison (*n* = 5 cells). A biocytin cell fill was also recovered for one recorded VEN (Fig. 5c), with confirmed layer 5 localization based on soma location (1.7 mm from the pial surface of the slice) in the DAPI stain. Consistent with previous reports, this cell displayed the expected large spindle-shaped morphology with large caliber bipolar dendrites that extended into layer 6 (descending trunk), as well as towards the pial surface into upper layer 3 (ascending trunk). Dendritic branching was very simple, but with notable short and wispy lateral branches concentrated proximal to the soma. The axon could not be readily distinguished from these finer dendrites. The fill quality was not sufficient to identify clear dendritic spines; however, these recorded VENs appear to have a lower spine density than recorded pyramidal cells, consistent with previous reports based on Golgi staining^3^.

We observed marked differences in the suprathreshold response of putative VENs vs. neighboring pyramidal neurons in response to 1s current injection steps (Fig. 5d–g). Specifically, VENs produced fewer action potentials in response to a given level of current injection. This difference may be related to differences in spike timing during a train of action potentials; putative VENs displayed higher variability in spike timing and greater spike frequency accommodation than neighboring pyramidal neurons. All putative VENs displayed brief pauses and prominent subthreshold membrane oscillations during sustained firing. Although this result was not statistically significant (*p* > 0.05), the differences in average input resistance of the putative VENs (61 ± 14 Mohms, mean ± standard error [SE]) compared with neighboring pyramids (113 ± 25 Mohm, mean ± SE) may also contribute to differences in firing of these morphologically-distinct types. It is worth noting that the top marker for Exc *FEZF2 GABRQ*, namely *GABRQ*, is an ion channel that encodes the theta subunit of the GABA-A receptor, and that several other ion channels also show some enrichment in this cluster, albeit to a much lesser degree. While many small gene expression differences in ion channels could collectively influence the distinct electophysiological properties we have described, the functional impact of any given ion channel will be an important topic for future study.

## Discussion

To determine if VENs represent a discrete transcriptionally-defined cell type, we applied snRNA-seq to classify neurons in FI layer 5 and carried out cross-species homology mapping to make predictions about VEN cellular phenotypes that are difficult to measure in human tissues. We define 13 excitatory neuron types and show that VENs can be localized to a single transcriptomic cell type (Exc *FEZF2 GABRQ*), but this cell type also includes cells with fork and pyramidal morphologies. This approach identified many novel and selective marker genes of VENs and other excitatory neuron types that will facilitate better identification and study of these populations in situ. However, consistent with all published studies to date, we do not find a molecular signature that can distinguish VENs from transcriptionally similar fork or pyramidal neurons that comprise the Exc *FEZF2 GABRQ* type. Several explanations for this inability to distinguish VENs from closely-related cells on the basis of gene expression signatures are possible. First, it is possible that none of our sorted putative ET nuclei were from VENs; however, this is highly unlikely given the high frequency of VENs expressing ET markers in our validation studies (~25%). Second, these morphologically-defined types may not be molecularly distinct in the adult, but rather could represent a spectrum of morphologies established during development within a broader excitatory cell class. Alternatively, the current study may have lacked the power to discriminate closely related VEN and pyramidal neuron types from the 23 total neurons in the putative ET cluster. Supporting this latter idea, greater diversity of ET neurons is seen in mouse where these cells are more abundant and can be selectively enriched^30,32^, including one type projecting predominantly to myelencephalon and others targeting additional subcortical areas^30^. Further studies using higher-throughput snRNA-seq technologies will be required to definitively answer this question, but it is clear that VENs, fork cells and a subset of pyramidal cells are transcriptomically similar to one another.

Homology mapping to mouse predicts that VENs are likely subcortically-projecting ET neurons. Prior studies in mouse demonstrate a robust division between locally-projecting (IT) and subcortically-projecting (ET) neurons based on gene expression^30^ as well as electrophysiology^33,34^. Alignment of human FI data with mouse cortical scRNA-seq data shows that Exc *FEZF2 GABRQ* is homologous to ET neurons in mouse VISp and ALM^30^. In addition, VENs express transcription factors required for the generation of subcortically-projecting neurons, such as *FEZF2*^29^, but do not express transcription factors associated with corticothalamic or callosal projections^28^. Lastly, a study in rhesus monkey proposed that VENs primarily project to distant subcortical regions, including the parabrachial nucleus of dorsolateral pons and the midbrain periaqueductal gray^27^. Together, our findings and those of previous reports^27,28^ support the hypothesis that VENs project to deep subcortical structures. However, VEN projections might not be restricted to ET targets as tract-tracing shows that some VENs project to both ipsilateral and contralaterial cortical targets, potentially including VEN populations within homologous structures of the contralaterial hemisphere^27^. Matching of a transcriptomic cluster that includes VENs and fork cells in human to ET types in mouse that do not have these distinct morphologies suggests that, like rosehip neurons^44^ and interlaminar astrocytes^32^, VENs and fork cells may represent morphological diversification of an evolutionarily conserved cell type. Furthermore, we recently used homology mapping in human MTG and identified a putative ET type homologous to mouse ET types^32^. This MTG ET type aligns to the FI ET type, and much of the molecular signature is shared between these putative ET types in human FI and MTG. However, many genes are expressed selectively by the FI type that are distinct from the MTG type, suggesting potential regional specialization of these excitatory neurons.

Selective loss of VENs and fork cells in FI and ACC has been proposed to contribute to several neuropsychiatric disorders characterized by social-emotional deficits^16,18–21,48^. Many of these disorders show dysfunction of the salience network^48^, which has key nodes in these same brain regions^49^ and coordinates the brain’s responses to behaviorally-relevant stimuli^50^, suggesting a direct link between VEN loss and dysfunction. Additionally, the salience network has functional connectivity in several subcortical areas including parts of amygdala, striatum, dorsomedial thalamus, and substantia nigra^49^, consistent with the predicted projection targets of VENs. However, our results suggest the possibility that bvFTD and other neuropsychiatric disorders targeting FI might result from loss of ET neurons more generally, rather than exclusive loss of VENs and fork cells. The novel markers identified here for ET neurons and other excitatory types provide opportunities for a refined analysis of disease-related loss of excitatory neurons. Importantly, despite the lack of VENs in rodent brains, mouse models of bvFTD are surprisingly effective at recapitulating histopathological^51^ and behavioral^52^ impairments reported in humans, suggesting that additional cell types are likely affected or that rodent has a homologous type to VENs that, despite different morphology, has similar circuit function.

A major challenge in understanding human brain cellular and circuit function is a paucity of tools, techniques, and tissue. However, techniques for physiological and morphological analysis using in vitro slice preparations and patch clamp physiology work robustly on human tissue from neurosurgical resections^44,53–58^. Although it is exceedingly rare for tissue to be removed from regions like FI and ACC during such surgeries, the instances in which such specimens can be collected for research purposes represent rare opportunities to collect highly valuable data in the spirit of case studies in disease, where even sparse data can provide important observations and generate testable hypotheses. From the singular such specimen collected in more than three years, we demonstrate that neurons with VEN-like morphologies in layer 5 of human insula can be targeted and functionally characterized. Further, these putative VENs exhibit distinctive intrinsic physiological properties compared to neighboring pyramidal neurons in the same brain region. Given the small number of neurons recorded from L5 insula of a single patient, it will be important to replicate these findings, should such rare opportunities arise in the future. Nonetheless, these data represent the first reported patch clamp recordings from putative VENs in the human insula, and our findings are consistent with the hypothesis that VENs represent a functionally specialized cell type. Further evidence will be necessary to establish the exact contributions of this cell type to human brain function in health and disease.

It is essential to find experimental strategies to understand the specifics of the human brain, particularly for cell types affected by diseases that are not well modeled in widely used experimental organisms. New technological advances built on the transcriptomic approach to cell type classification promise to accelerate progress on functional analyses of human neuron types. Patch-seq allows the combination of electrophysiological, transcriptomic and morphological analysis^59,60^, which can in principle be applied to human brain slice studies over extended time frames with recent advances in culturing of human ex vivo brain tissue^53^. Furthermore, novel viral tools enable cell type-specific genetic targeting in this system^61^, and application of enhancers for ET cells, such as the *Fam84b* enhancer that labels mouse ET cells with >90% specificity^62^, represents a potential avenue for labeling and studying the properties of VENs in ex vivo human brain tissue. Such studies can help to further refine our understanding of the characteristics of VENs, potentially providing information about their local connectivity and teasing out subtle gene expression differences between putative ET cells with spindle, fork, and pyramidal morphologies.

## Methods

### Ethical compliance

De-identified postmortem human brain tissue was collected by the San Diego Medical Examiner’s office and provided to the Allen Institute for Brain Science after obtaining permission from decedent next-of-kin. The Western Institutional Review Board (WIRB) reviewed the use of de-identified postmortem brain tissue for research purposes and determined that, in accordance with federal regulation 45 CFR 46 and associated guidance, the use of and generation of data from de-identified specimens from deceased individuals did not constitute human subjects research requiring institutional review board (IRB) review. Postmortem tissue collection was performed in accordance with the provisions of the United States Uniform Anatomical Gift Act of 2006 described in the California Health and Safety Code section 7150 (effective 1/1/2008) and other applicable state and federal laws and regulations.

Tissue procurement from neurosurgical donors was performed outside of the supervision of the Allen Institute for Brain Science at local hospitals, and tissue was provided to the Allen Institute for Brain Science under the authority of the IRB of each participating hospital. A hospital-appointed case coordinator obtained informed consent from donors prior to surgery. Tissue specimens were de-identified prior to receipt by Allen Institute personnel. The specimen collected for this study was apparently non-pathological tissue removed during the normal course of tumor surgery. The tissue specimen collected was determined to be non-essential for diagnostic purposes by medical staff and would have otherwise been discarded.

### Postmortem tissue donors

Postmortem tissue donors were prescreened for history of neuropsychiatric disorders, neuropathology, and infectious disease (HIV, Hepatitis B, Hepatitis C), and postmortem blood samples were sent for routine serology and toxicology testing. Specimens were further screened for RNA quality and had an RNA integrity number (RIN) ≥ 7. Tissues used for RNA-sequencing in this study were from two control Caucasian male donors who died from cardiovascular-related issues, aged 50 (H200.1025) and 54 (H200.1030) years, as previously described^44^.

### Tissue processing and isolation of nuclei

Whole postmortem brain specimens were processed as previously described^32,44^. For RNA-sequencing experiments, frontoinsula (FI) was identified on frozen cortex slabs of interest, and the region of interest was removed and vibratome sectioned at 500 µm intervals^32,44^ (Fig. 1). Layer 5 was microdissected from vibratome sections stained with fluorescent Nissl and nuclei were isolated from microdissected tissue pieces using Dounce homogenization. Mouse monoclonal anti-NeuN antibody (EMD Millipore, MAB377) was applied to nuclei preparations followed by secondary antibody staining (goat anti-mouse Alexa Fluor 594, ThermoFisher), and single-nucleus sorting was carried out on a BD FACSAria Fusion instrument (BD Biosciences) using a 130 µm nozzle following a standard gating procedure (Supplementary Fig. 1)^32,44^. Approximately 10% of nuclei were intentionally sorted as NeuN–negative to capture non-neuronal cell types. Single nuclei were sorted into 96-well PCR plates (ThermoFisher Scientific) containing 2 µL of lysis buffer (0.2% Triton-X 100, 0.2% NP-40 (Sigma Aldrich), 1 U/µL RNaseOUT (ThermoFisher Scientific), PCR-grade water (Ambion), and ERCC spike-in synthetic RNAs (Ambion). 96-well plates were snap frozen and stored at −80 °C until use. Positive controls were pools of 10 nuclei, 10 pg total RNA, and 1 pg total RNA.

### cDNA and sequencing library preparation

Single nucleus cDNA libraries were prepared using Smart-seq2 with minor modifications as follows: Protoscript II (New England Biolabs) was used for reverse transcription, the final dilution of ERCCs in the reverse transcription reaction was 1:55 million, the template switching oligonucleotide was 5′-biotinylated, and 21 PCR cycles were used for cDNA amplification^44^. Sequencing libraries were prepared using Nextera XT (Illumina) with input cDNA at 250 pg per reaction; reactions were carried out at 1/4 the volume recommended by the manufacturer with a 10 min tagmentation step. Libraries were sequenced on a HiSeq 4000 instrument (Illumina) using 150 bp paired-end reads.

### RNA-seq data processing

SnRNA-seq data were processed and analyzed as previously described^38,44^. Briefly, following demultiplexing of barcoded reads generated on the Illumina HiSeq platform, the amplification (cDNA and PCR) and sequencing primers (Illumina) and the low-quality bases were removed using Trimmomatic 0.35 software^63^. Trimmed reads were mapped to the human reference genome, version GRCh38 (Ensembl), guided by the version 21 annotations obtained from the GENCODE repository. RSEM 1.2.31^64^, TopHat 2.1.1, and Cufflinks 2.2.1^65^ were used to quantify transcript expression at the transcriptome (exon) and whole-genome (exon plus intron) levels, respectively. Software packages fastQC 0.10.1 (http://www.bioinformatics.babraham.ac.uk/projects/fastqc/), FASTX 0.0.14 (http://hannonlab.cshl.edu/fastx_toolkit/download.html), RSeQC 2.6.1^66^, and RNA-seq-QC 1.1.8^67^ were used to generate various sequence and alignment quality metrics used for classifying sample quality. A novel pipeline (SCavenger, J.M., unpublished) was created to automate execution across statistical analysis tools, integrate preformatted laboratory and clustering metrics, and calculate new statistics specific to biases identified in the single-nuclei lab and sequence preparation protocol.

### RNA-seq quality control

To remove data from low-quality samples before downstream analysis, we implemented a random forest machine-learning classification approach as previously described^44,68^. The overall workflow for sample quality classification and filtering was to (i) establish a training set using a representative subset of samples, (ii) collect a series of 108 quality control metrics (for example, percent unique reads, percent reads surviving trimming, transcript isoform counts) spanning both the laboratory and data analysis workflows as model features, (iii) use these training data and quality control metrics to build a classification model using the random forest method, and (iv) apply the model to the entire dataset for quality classification and data filtering.

The random forest quality control model was then applied to the data and final quality Pass-Fail classifications were determined. A Pass confidence cutoff of 0.6 or greater was used to select single-nuclei data for downstream analysis. Using this random forest model applied to the entire layer 5 dataset, 78% of 1118 single-nuclei samples passed quality control (*N* = 876). For these Pass samples, the average number of reads after trimming was 16,715,521 ± 20,434,739, the number of ERCC transcripts detected was 41.78 ± 4.79 out of 92, and the average number of genes detected across all passing nuclei at FPKM > 1 was 5584 ± 2004, giving an average coverage of 2174 reads per human gene detected. Additional summary statistics (grouped by donor or cluster) for nuclei passing QC and included in the analysis are shown in Supplementary Fig. 1.

### Gene expression calculation

For each nucleus, expression levels were estimated based on the scaled coverage across each gene. Specifically, bam files were read into R using the readGAlignmentPairs function in the GenomicAlignments library, and genomic coverage was calculated using the coverage function in GenomicRanges^69^. All genes in GENCODE human genome GRCh38, version 21 (Ensembl 77; 09-29-2014) were included, with gene bounds defined as the start and end locations of each unique gene specified in the gtf file (https://www.gencodegenes.org/releases/21.html). Total counts for each gene (including reads from both introns and exons) were estimated by dividing total coverage by twice the read length (150 bp, paired end). Expression levels were normalized across nuclei by calculating counts per million (CPM).

### Clustering nuclei

Nuclei and cells were grouped into transcriptomic cell types using an iterative clustering procedure as described in Boldog et al.^44^. Briefly, intronic and exonic read counts were summed, and log2-transformed expression (CPM + 1) was centered and scaled across nuclei. Differentially expressed genes were selected while accounting for gene dropouts, and principal components analysis (PCA) followed by *t*-distributed stochastic neighbor embedding (t-SNE)^70^ was used to reduce dimensionality. Nearest-neighbor distances between nuclei were calculated, and segmented linear regression (*segmented* R package) was applied to estimate the distribution breakpoint to help define the distance scale for density clustering. The statistical significance of the separation of clusters identified by density clustering was evaluated with the R package sigclust^71^, which compares the distribution of nuclei to the null hypothesis that nuclei are drawn from a single multivariate Gaussian. Iterative clustering was used to split nuclei into subclusters until the occurrence of one of four stop criteria: (i) fewer than 6 nuclei in a cluster (because it cannot be split due a minimum cluster size of 3), (ii) no significantly variable genes, (iii) no significantly variable principal components, or (iv) no significant subclusters.

To assess the robustness of clusters, the iterative clustering procedure described above was repeated 100 times for random subsamples of 80% of nuclei. A co-clustering matrix was generated that represented the proportion of clustering iterations in which each pair of nuclei was assigned to the same cluster. Average-linkage hierarchical clustering was applied to this matrix, followed by dynamic branch cutting (R package WGCNA) with cut heights ranging from 0.01 to 0.99 in steps of 0.01. A cut height resulting in 25 clusters was selected to balance cohesion (average within cluster co-clustering) and discreteness (average between cluster co-clustering) across clusters. Finally, gene markers were identified for all cluster pairs, and clusters were merged if they lacked binary markers (gene expressed in >50% nuclei in first cluster and <10% in second cluster) with average CPM > 1. Clusters were marked as outliers and excluded from analysis if they contained lower quality nuclei based on QC metrics or expression of mitochondrial genes.

To retain only a high confidence set of clusters, we used two strict quality control steps. One primary quality control step was to exclude clusters containing only cells from a single donor. This filtering decreases the chances that identified cell types are due to low tissue quality or batch effects. Second, we removed clusters containing primarily low quality cells due to poor RNA quality. These clusters were identified as having meta-data values more than three standard deviations below the mean. For example, nuclei from these excluded clusters had a distinct expression signature of approximately 100 non-coding genes including miRNAs and pseudogenes that had low expression in other passing nuclei. In addition, the clusters defined as outlier clusters all had lower median pass scores as defined using the machine-learning classifier described above, suggesting that our initial threshold may have been too lenient. Around half the 315 excluded nuclei were removed for each of these two reasons, leaving a total of 561 nuclei in the passing clusters presented in this analysis. A slightly modified version of this entire clustering process is available in the scrattch.hicat R library on GitHub (https://github.com/AllenInstitute/scrattch.hicat).

Cluster names were defined using an automated strategy as previously described^32^. Briefly, clusters were assigned to the major classes interneuron, excitatory neuron, microglia, astrocyte, oligodendrocyte precursor, or oligodendrocyte based on maximal median cluster CPM of *GAD1, SLC17A7, C3, AQP4, CSPG4, or OPALIN*, respectively. Clusters were then assigned a subclass marker, defined by maximal median CPM of *LAMP5, VIP, SST, PVALB, LHX6, LINC00507, RORB, THEMIS, FEZF2, CTGF, C3, FGFR3, CSPG4*, or *OPALIN*. Finally, clusters in all major classes that contained more than one cluster were assigned a cluster-specific marker gene. These marker genes had the greatest difference in the proportion of expression (CPM > 1) with a cluster compared to all other clusters regardless of mean expression level. In some cases the most specific marker gene was the subclass marker (*SST* and *VIP*).

### Scoring cluster marker genes

Many genes were expressed in the majority of nuclei in a subset of clusters. A marker score (beta) was defined for all genes to measure how binary expression was among clusters, independent of the number of clusters labeled. labeled. First, the proportion (*xi*) of nuclei in each cluster that expressed a gene above background level (CPM > 1) was calculated. Then, scores were defined as the squared differences in proportions normalized by the sum of absolute differences plus a small constant (ε) to avoid division by zero. Scores ranged from 0 to 1, and a perfectly binary marker had a score equal to 1.

$$\frac{{\mathop {\sum }\nolimits_{i = 1}^n \mathop {\sum}\nolimits_{j = 1}^n {\left( {x_i - x_j} \right)^2} }}{{\mathop {\sum }\nolimits_{i = 1}^n \mathop {\sum}\nolimits_{j = 1}^n {|x_i - x_j| + {\it{\epsilon }}} }}.$$

### Enrichment marker genes

Genes were defined as enriched in Exc *FEZF2 GABRQ* if they met the following criteria: (1) they were expressed in at least half the cells in Exc *FEZF2 GABRQ*, (2) they were expressed in fewer than half the cells in every other cluster, (3) they were expressed in at least 25% more cells in Exc *FEZF2 GABRQ* than in any cluster, and (4) the average expression in Exc *FEZF2 GABRQ* was at least two-fold higher than every other cluster. 30 genes met these criteria. Marker genes for the corresponding cluster in MTG (Exc L4-5 *FEZF2 SCN4B*) were identified the same way, but only genes that also were expressed in fewer than 30% of the cells in Exc *FEZF2 GABRQ* are show in Fig. 4a, b.

### Cluster dendrograms

Clusters were arranged by transcriptomic similarity based on hierarchical clustering. First, the average expression level of the top 1200 scoring cluster marker genes (highest beta scores, as above) was calculated for each cluster. A correlation-based distance matrix ($D_{xy} = \frac{{1 \,-\, \rho \left( {x,y} \right)}}{2}$) was calculated, and complete-linkage hierarchical clustering was performed using the “hclust” R function with default parameters. The resulting dendrogram branches were reordered to show inhibitory clusters followed by excitatory clusters, with larger clusters first, while retaining the tree structure. Note that this measure of cluster similarity is complementary to the co-clustering separation described above. For example, two clusters with similar gene expression patterns but a few binary marker genes may be close on the tree but highly distinct based on co-clustering.

### Gene expression visualization

Gene expression (CPM) was visualized using heat maps and violin plots, which both show genes as rows and nuclei as columns, sorted by cluster. Heat maps display each nucleus as a short vertical bar, color-coded by expression level (blue = low; red = high), and clusters were ordered as described above. The distributions of marker gene expression across nuclei in each cluster were represented as violin plots, which are density plots turned 90 degrees and reflected on the *y*-axis. Black dots indicate the median gene expression in nuclei of a given cluster; dots above *y* = 0 indicate that a gene is expressed in more than half of the nuclei in that cluster.

### Colorimetric in situ hybridization

Information about postmortem tissue donors and methods used for colorimetric in situ hybridization (ISH) is available from the Allen Human Brain Atlas documentation at http://human.brain-map.org/.

### Multiplex fluorescent in situ hybridization (FISH)

Human tissue specimens used for RNAscope mFISH came from a cohort of neurosurgical resection and postmortem tissues that included donors used for snRNA-seq. Fresh-frozen tissues were sectioned at 14–16 μm onto Superfrost Plus glass slides (Fisher Scientific). Sections were dried for 20 min at −20 °C and then vacuum sealed and stored at −80 °C until use. The RNAscope multiplex fluorescent v1 kit was used per the manufacturer’s instructions for fresh-frozen tissue sections (ACD Bio), except that fixation was performed for 60 min in 4% paraformaldehyde in 1X PBS at 4 °C and protease treatment was shortened to 10 min. Positive controls used to assess RNA quality in tissue sections were either a set from ACD Bio (*POLR2A, PPIB, UBC*, #320861) or a combination of *SLC17A7, VIP*, and *GFAP*. Sections were imaged using either a 40X or 60X oil immersion lens on a Nikon TiE fluorescent microscope equipped with NIS-Elements Advanced Research imaging software (version 4.20). For all RNAscope mFISH experiments, positive cells were called by manually counting RNA spots for each gene. Cells were called positive for a gene if they contained ≥5 RNA spots for that gene. Lipofuscin autofluorescence was distinguished from RNA spot signal based on the larger size of lipofuscin granules and broad fluorescence spectrum of lipofuscin.

### Dual chromogenic in situ hybridization

Dual chromogenic in situ hybridization (dISH) was performed using the RNAscope 2.5 HD Duplex Assay Kit (ACD Bio) per the manufacturer’s protocol. Experiments were performed using fresh-frozen tissues sectioned at 16–25 μm onto Superfrost Plus glass slides (Fisher Scientific) and sections were counterstained with hematoxylin to visualize nuclei.

### Scoring of morphological types

Staining for the EXC *FEZF2 GABRQ* markers *ADRA1A* and *POU3F1* was carried out using dISH as described above. At least 3 sections from 5 individual human donors were used for morphological assessment and scoring. First, the total number of layer 5 cells positive for both *ADRA1A* and *POU3F1* was determined for each donor. Then, the morphology of each double-positive cell was assessed and scored as either pyramidal (cell body round to pyramidal in shape and wider than tall), VEN (cell body elongated, spindle-shaped and taller than wide) and uncharacterized (lacking definitive morphological features perhaps due to bisection of cells during sectioning). The proportion of cells in each morphological type was then calculated as a fraction of the total number of *ADRA1A* and *POU3F1* double-positive cells. Cells were called positive for a gene if they contained ≥5 RNA spots for that gene.

### Quantification of putative extratelencephalic (ET) neurons

The fraction of putative ET neurons in FI and MTG was estimated using both mFISH and snRNA-Seq. For mFISH estimates, the total numbers of *SLC17A7*+, *POU3F1*+ and *SLC17A7*+, *POU3F1*− cells in layer 5 were quantified in at least 3 sections per donor (*n* = 3 donors for both FI and MTG). The percentage of putative ET cells (*SLC17A7*+, *POU3F1*+) was then calculated as a fraction of the total number of *SLC17A7*+ cells in layer 5. SnRNA-seq estimates were made by taking the total number of neurons mapping to the relevant ET cluster (Exc *FEZF2 GABRQ* and Exc L4-5 *FEZF2 SCN4B* in FI and MTG, respectively) and dividing by the total number of excitatory neurons collected in layer 5 dissections.

### Cross-species data integration

To assess cross species cell type homology, excitatory cells (mouse) or nuclei (human) collected from human FI (these data), human MTG^32^, mouse VISp, and mouse ALM^30^ were compared. Log2-transformed CPM of intronic plus exonic reads was used as input for all four datasets. Including exonic reads increased experimental differences due to measuring whole cell vs. nuclear transcripts, but this was out-weighed by improved gene detection. To the extent possible, a matched subset of cells was included as input to Seurat. In human MTG, we included all cells dissected from layers 4 or 5 that were mapped to excitatory clusters with at least 10 total cells from layer 5, including up to 50 randomly sampled cells per cluster (for a total of 616 nuclei); cells from layer 4 were included since FI does not contain a layer 4. In mouse VISp and ALM, cells were grouped by subclass (rather than cell type) and we selected 100 random cells per subclass (for a total of 700 in ALM, which does not contain layer 4, and 800 in VISp). All genes that could be matched between data sets, except a set of sex and mitochondrial genes, were considered.

These data sets were assembled into an integrated reference using Seurat V3 (https://satijalab.org/seurat/)^41,42^ following the tutorial for Integration and Label Transfer and using default parameters for all functions, except when they differed from those used in the tutorial. Seurat is an R package which can take multiple single-cell RNA-seq data sets as input, and then perform normalization, alignment (also called integration), and clustering of all of these data together. When aligning multiple data sets, Seurat identifies shared pairwise correspondences of cells across data sets and uses these anchors to transfer information from one set to another for alignment. We chose to apply this method for its ease of use, but also find that our result is robust to choice of method.

More specifically, we first selected the union of the 2000 most variable genes in each data set (using FindVariableFeatures with method = “vst”). Next, we projected this data sets into subspace based on common correlation structure using canonical correlation analysis (CCA) followed by L2 normalization, and found integration anchors (cells that are mutual nearest neighbors between data sets) in this subspace. Each anchor is weighted based on the consistency of anchors in it’s local neighborhood, and these anchors were then used as input to guide data integration (or batch-correction), as proposed previously^72^. We then scaled the data, reduced the dimensionality using principal component analysis, and visualized the results with Uniform Manifold Approximation and Projection (UMAP)^73^. We defined homologous cell types by constructing a shared nearest neighbor (SNN) graph on the integrated data sets based on the Jaccard similarity of the 10 nearest neighbors of each sample. Louvain community detection was run to identify clusters that optimized the global modularity of the partitioned graph. Data set clusters are grouped based on the maximal fraction of cells in these Seurat-assigned cluster, which were nearly perfectly aligned for most subclasses, including ET. Changes in parameters did not change the integration of cluster Exc *FEZF2 GABRQ* with mouse ET clusters.

In addition, we performed correlation-based cell type matching of human FI to mouse VISp and ALM (independently) and show that human and mouse ET cells align. For each gene, we scaled the data such that the maximum log2(CPM+1) across human cells and across mouse cell type means was set to 1. We then selected the 75, 100, 150, 250, or 500 most depleted and most enriched genes in the mouse clusters and used these genes to calculate the Pearson correlation between each human FI cell and each mouse cell type, assigning each human cell to the mouse cell type with maximal correlation. In all cases, we found that the 23 human cells in the Exc *FEZF2 GABRQ* cluster were assigned to the mouse ET clusters.

### Electrophysiology

Electrophysiological experiments were performed as reported previously^54^. Briefly, the surgical tissue specimen obtained from the insula was sectioned into 300 μm thick slices using a Compresstome VF-200 (Precisionary Instruments) in a solution composed of (in mM): 92 with N-methyl-D-glucamine (NMDG), 2.5 KCl, 1.25 NaH_2_PO_4_, 30 NaHCO_3_, 20 4-(2-hydroxyethyl)-1-piperazineethanesulfonic acid (HEPES), 25 glucose, 2 thiourea, 5 Na-ascorbate, 3 Na-pyruvate, 0.5 CaCl_2_•4H_2_O and 10 MgSO_4_•7H_2_O. After warming for 10 min in the same solution, slices were transferred to a holding chamber containing 92 NaCl, 2.5 KCl, 1.25 NaH_2_PO_4_, 30 NaHCO_3_, 20 HEPES, 25 glucose, 2 thiourea, 5 Na-ascorbate, 3 Na-pyruvate, 2 CaCl_2_•4H_2_O and 2 MgSO_4_•7H_2_O. Slices were submerged in a recording chamber continually perfused with artificial cerebrospinal fluid (aCSF) consisting of 119 NaCl, 2.5 KCl, 1.25 NaH_2_PO_4_, 24 NaHCO_3_, 12.5 glucose, 2 CaCl_2_•4H_2_O and 2 MgSO_4_•7H_2_O and were viewed with an Olympus BX51WI microscope equipped with infrared differential interference contrast optics and a ×40 water immersion objective.

Whole cell somatic recordings were acquired using a Multiclamp 700B amplifier and PClamp 10 data acquisition software (Molecular Devices). Electrical signals were digitized at 20–50 kHz and filtered at 2–10 kHz. The pipette solution contained 130 K-gluconate, 4 KCl, 10 HEPES, 0.3 EGTA, 10 Phosphocreatine-Na_2_, 4 Mg-ATP, 0.3 Na_2_-GTP, 0.5% biocytin and .020 Alexa 594. Pipette capacitance was compensated and the bridge was balanced throughout the recording.

Data were analyzed using custom analysis scripts written in Igor Pro (Wavemetrics). All measurements were made at resting potential. FI curves were constructed by measuring the number of action potentials elicited by 1s long current injections of increasing amplitude (Δ50 pA). Spike frequency accommodation was determined from the current injection yielding 10 ± 2 spikes and was calculated as the ratio of the last to the second interspike interval. The coefficient of variation of spike times was calculated from the same sweep.

### Reporting summary

Further information on research design is available in the Nature Research Reporting Summary linked to this article.

## Supplementary information

Supplementary Information

Peer Review

Reporting Summary

## Data Availability

Raw and aligned data have been registered with dbGaP (https://www.ncbi.nlm.nih.gov/projects/gap/cgi-bin/study.cgi?study_id=phs001791.v1.p1) and have been deposited in the NeMO archive (https://nemoarchive.org/) for controlled access, as soon as such functionality in NeMO becomes available. In addition, aligned (count) data is available on GitHub (https://github.com/AllenInstitute/L5_VEN).

## Code availability

Custom R code and count data used to generate transcriptomics related figures can be downloaded from https://github.com/AllenInstitute/L5_VEN.
