# Supplementary material for: Transcriptomic evidence that von Economo neurons are regionally specialized extratelencephalic-projecting excitatory neurons
Source: Nat Commun. 2020 Mar 3;11:1172. doi: 10.1038/s41467-020-14952-3 (PMC7054400; doi:10.1038/s41467-020-14952-3)
Supplement: Supplementary file 1 — Supplementary Information [file 41467_2020_14952_MOESM1_ESM.pdf]

Supplementary Information

Transcriptomic evidence that von Economo neurons are regionally specialized extratelencephalic-projecting excitatory neurons

Hodge et al.

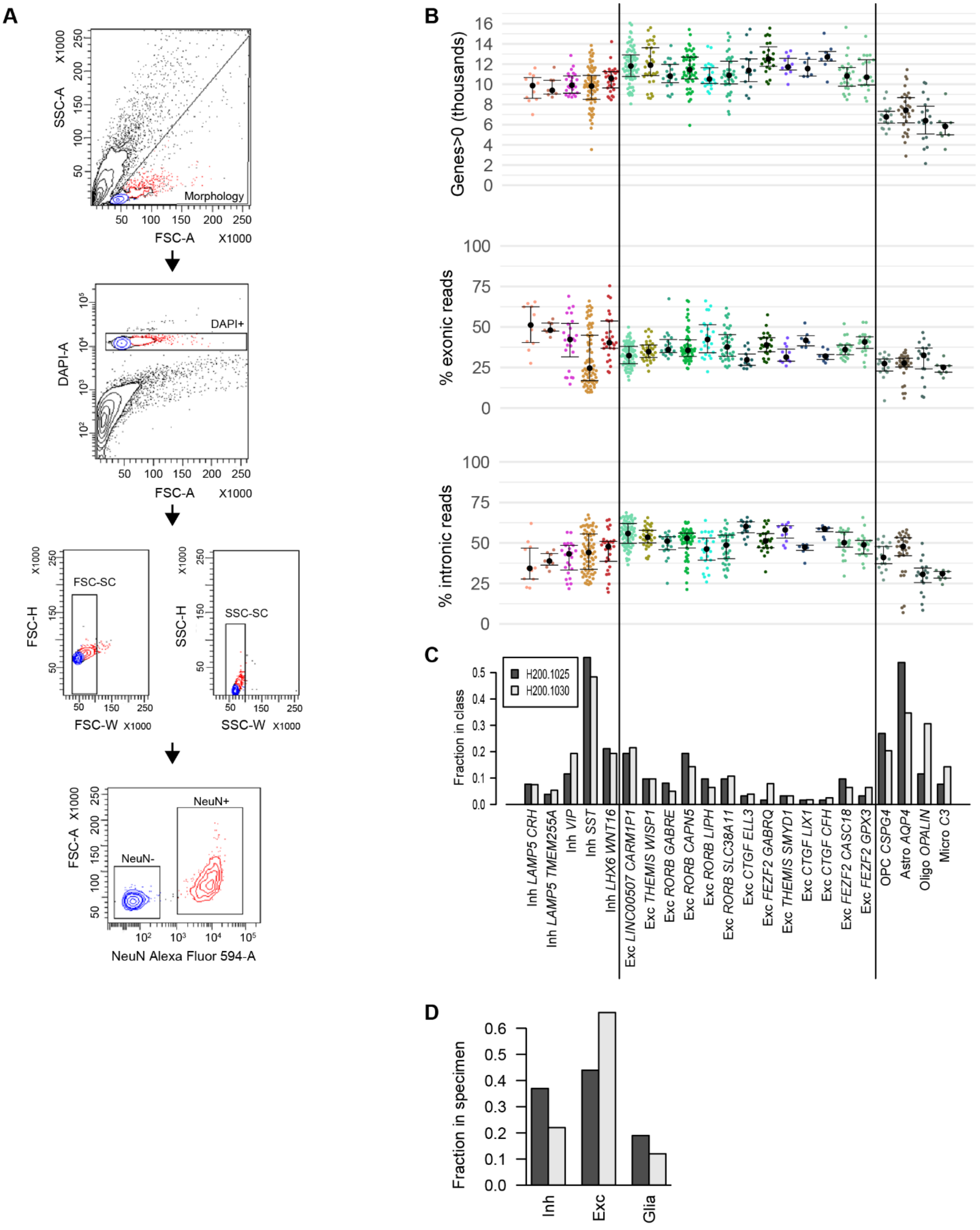
A
SSC-A X1000
FSC-A X1000
Morphology
DAPI-A
DAPI+
FSC-H X1000
FSC-SC
FSC-W X1000
SSC-H X1000
SSC-SC
SSC-W X1000
FSC-A X1000
NeuN-
NeuN+
NeuN Alexa Fluor 594-A
B
Genes>0 (thousands)
% exonic reads
% intronic reads
C
H200.1025
H200.1030
Fraction in class
Inh LAMP5 CRH
Inh LAMP5 TMEM255A
Inh VIP
Inh SST
Inh LHX6 WNT16
Exc LINC00507 CARM1P1
Exc THEMIS WISP1
Exc RORB GABRE
Exc RORB CAPN5
Exc RORB LIPH
Exc RORB SLC38A11
Exc CTGF ELL3
Exc FEZF2 GABRQ
Exc THEMIS SMYD1
Exc CTGF LIX1
Exc CTGF CFH
Exc FEZF2 CASC18
Exc FEZF2 GPX3
OPC CSPG4
Astro AQP4
Oligo OPALIN
Micro C3
D
Fraction in specimen
Inh
Exc
Glia

Supplementary Figure 1. Summary of sorting strategy and cluster statistics. (A) Standard fluorescence activated-cell sorting (FACS) gating scheme for collection of NeuN-positive and NeuN-negative nuclei. **(B)** Scatter plots plus median and interquartile interval of three QC metrics grouped and colored by cluster. Median gene detection was highest among excitatory neuron types (with Exc *FEZF2 GABRQ* the highest overall), lower among inhibitory neuron types, and significantly lower among non-neuronal types. All clusters had a large fraction of both exonic and intronic reads, with high cell type dependencies on these measures. **(C-D)** Bar plots showing that the fraction of nuclei from each cell type is relatively consistent between the two donors, when scaled by the total number of inhibitory, excitatory, and non-neuronal nuclei collected from each donor, but that a disproportionately smaller fraction of excitatory nuclei collected from donor H200.1025 passed QC compared both with donor H200.1030 and with expectations. Vertical lines separate major cell classes. Each bar represents a single point.

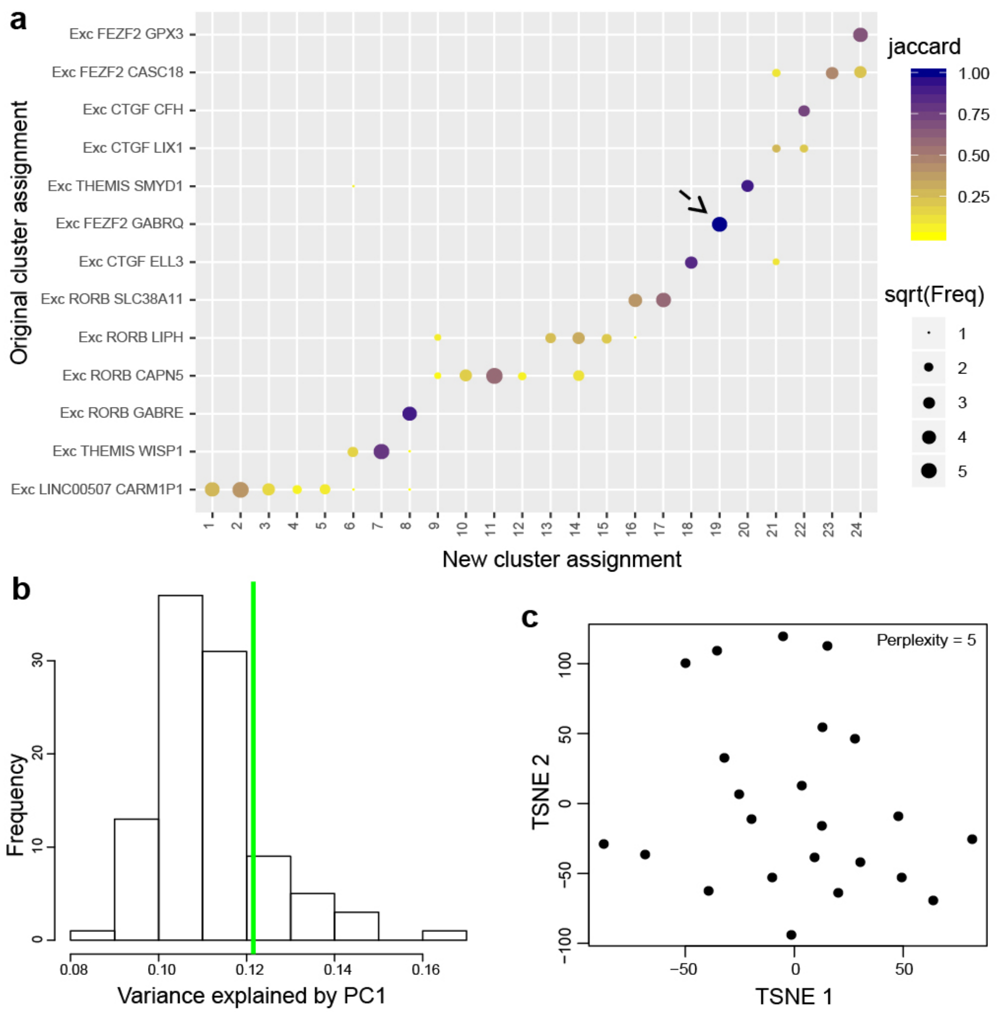

Supplementary Figure 2. Alternative clustering strategies fail to identify subdivisions of Exc *FEZF2 GABRQ*. **(A)** Reclustering of excitatory nuclei with more lenient parameters increased the number of identified clusters from the 13 described in this manuscript (horizontal axis) to 24 clusters (vertical axis). Using these parameters, all 23 cells assigned to Exc *FEZF2 GABRQ* are assigned to a single cluster (19) with no confusion with other types (arrow). Circles show both the Jaccard distance (color) and number of assigned cells (size) in matched labels for the same cells across both clustering runs. **(B-C)** Supervised analysis of cells in the Exc *FEZF2 GABRQ* cluster using previously reported VEN markers (see **Supplementary Fig. 4**). **(B)** These genes do not provide a level of heterogeneity beyond what is found from an equal number of randomly selected genes, as measured by the fraction of variance explained by the first principal component (x-axis; P = 0.13; permutation test). **(C)** These genes failed to divide this cluster further using TSNE, as the first two TSNE coordinates (x and y axis, respectively) show only a single cluster of cells at various perplexity levels (perplexity = 5 shown above).

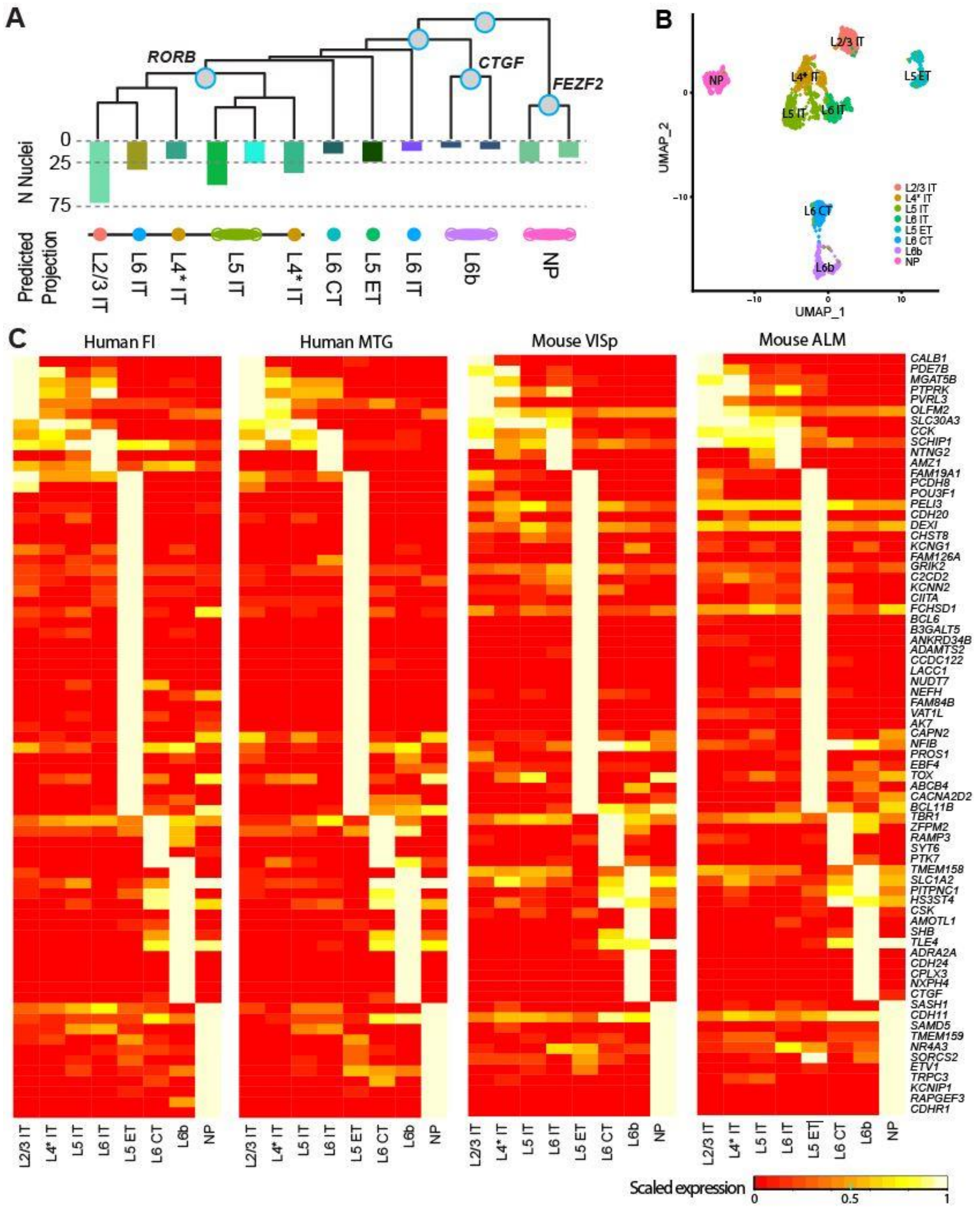

Supplementary Figure 3. Annotation of human excitatory types using mouse. **(A)** Eight Seurat clusters labeled based on expected cortical layer and projection target, as shown in **Figure 3B**. **(B)** Hierarchical representation of 13 excitatory cell types, as shown in **Figure 1B**, additionally labeled with their predicted projections from mouse (see **Figure 3**). **(C)** Heatmap showing 85 common marker genes for the eight matched clusters across all four data sets. These genes had high Pearson correlation of average Seurat cluster expression for all pairs of data sets (R>0.75) and high specificity, defined as the cumulative sum of the sorted average Seurat cluster expression, in each data set (S<3).

A

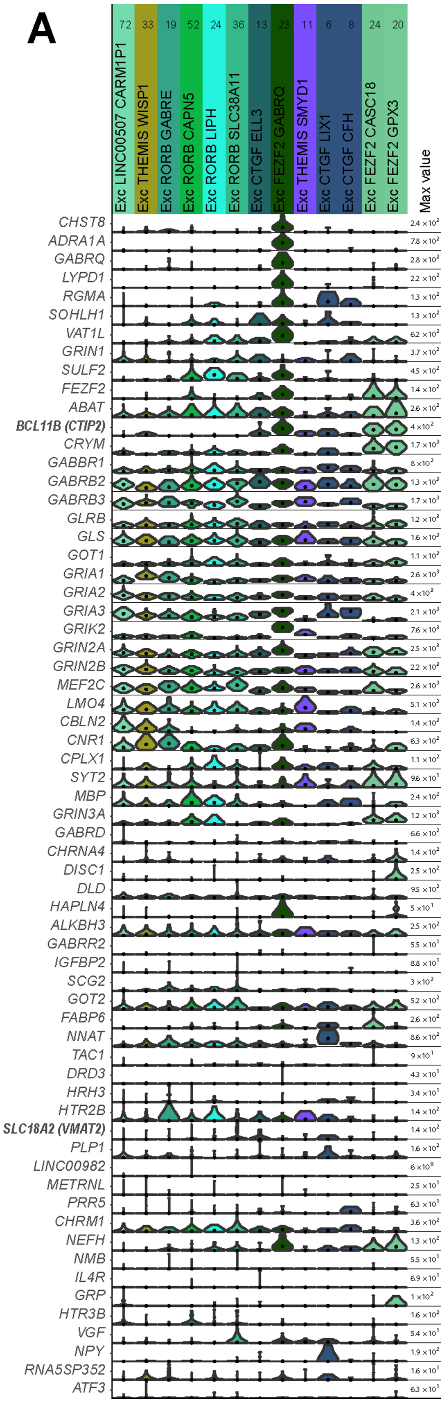

B

Gene	Allman 2005 (ICC)	Allman 2010 (IHC)	Stimpson 2011 (ICC)	Cobos 2013 (ISH/IHC/dFISH)	Dijkstra 2016 (ISH from ABA, IHC/ISH*)	Yang 2018 (LMD + RNA-seq, IHC/ISH/IF*)	
CHST8						X*	*
ADRA1A					X*		*
GABRQ					X*		*
LYPD1						X*	*
RGMA						X	
SOHLH1						X	
VAT1L						X*	*
GRIN1					e		*
SULF2						X*	
FEZF2				X		X	
ABAT					e		*
BCL11B				X			
CRYM					e		*
GABBR1					e		*
GABRB2					e		*
GABRB3					e		*
GLRB					e		*
GLS					e		*
GOT1					e		*
GRIA1					e		*
GRIA2					e		*
GRIA3					e		*
GRIK2					e		*
GRIN2A					e		*
GRIN2B					e		*
MEF2C						X	
LMO4				e			*
CBLN2					e		*
CNR1					e		*
CPLX1						X	
SYT2					e		*
MBP						X	
GRIN3A					e		
GABRD					e		
CHRNA4					e		
DISC1		X					
DLD					e		
HAPLN4						X	*
ALKBH3						X	
GABRR2					e		
IGFBP2						X	
SCG2					e		
GOT2					e		*
FABP6						X	
NNAT					e		
TAC1					e		
DRD3	X						
HRH3					e		
HTR2B	X						
SLC18A2					X*		
PLP1						X	
LINC00982						X	
METRNL						X	
PRR5						X	
CHRM1					e		
NEFH					e		*
NMB		X	X				
IL4R			X				
GRP		X					
HTR3B					e		
VGF					e		
NPY						X	
RNA5SP352						X	
ATF3			X				

Supplementary Figure 4. Expression of previously-reported VEN markers in human FI. **(A)** Violin plots showing expression of previously reported VEN marker genes per excitatory cluster. Around half of these genes are highly expressed in Exc *FEZF2 GABRQ*, but with highly varying levels of specificity. Genes are roughly ordered from most to least selective for Exc *FEZF2 GABRQ* in this study. Each row represents a gene, black dots show median gene expression within clusters, and the maximum expression value for each gene is shown on the right-hand side of each row. Gene expression values are displayed on a linear scale. **(B)** Specific evidence from previous publications about the specificity of the genes in **A**. Each column

corresponds to the listed publication, along with the methods used for identifying VEN markers (ICC, immunocytochemistry; IHC, immunohistochemistry; ISH, in situ hybridization; dFISH, double fluorescent ISH; ABA, Allen Brain Atlas; LMD, laser microdissection; IF, immunofluorescence). Marked boxes indicate that a given gene was found to be expressed in VENs in a given study (e = expressed in VENs, but not selective; X = selective in VENs as compared to surrounding pyramidal neurons; X* = validated as selective using at least one additional method, as indicated). Note that genes validated with multiple methods or in multiple studies tend to also be the most selective for Exc *FEZF2 GABRQ* in this study. Green asterisks indicate genes showing qualitative agreement between current and previous results (e.g. specific to the VEN cluster or expressed in the VEN cluster as previously reported). Previous studies targeted human frontoinsula, anterior cingulate cortex, or both.
