# Supplementary material for: Transcriptomic evidence that von Economo neurons are regionally specialized extratelencephalic-projecting excitatory neurons
Source: Nat Commun. 2020 Mar 3;11:1172. doi: 10.1038/s41467-020-14952-3 (PMC7054400; doi:10.1038/s41467-020-14952-3)
Supplement: Supplementary file 3 — Reporting Summary [file 41467_2020_14952_MOESM3_ESM.pdf]

nature research

Corresponding author(s): Ed Lein

Last updated by author(s): 01/23/2020

Reporting Summary

Nature Research wishes to improve the reproducibility of the work that we publish. This form provides structure for consistency and transparency in reporting. For further information on Nature Research policies, see Authors & Referees and the Editorial Policy Checklist .

Statistics

For all statistical analyses, confirm that the following items are present in the figure legend, table legend, main text, or Methods section.

n/a	Confirmed	
☐	☒	The exact sample size (*n*) for each experimental group/condition, given as a discrete number and unit of measurement
☐	☒	A statement on whether measurements were taken from distinct samples or whether the same sample was measured repeatedly
☐	☒	The statistical test(s) used AND whether they are one- or two-sided<br>*Only common tests should be described solely by name; describe more complex techniques in the Methods section.*
☒	☐	A description of all covariates tested
☐	☒	A description of any assumptions or corrections, such as tests of normality and adjustment for multiple comparisons
☐	☒	A full description of the statistical parameters including central tendency (e.g. means) or other basic estimates (e.g. regression coefficient) AND variation (e.g. standard deviation) or associated estimates of uncertainty (e.g. confidence intervals)
☒	☐	For null hypothesis testing, the test statistic (e.g. *F*, *t*, *r*) with confidence intervals, effect sizes, degrees of freedom and *P* value noted<br>*Give P values as exact values whenever suitable.*
☒	☐	For Bayesian analysis, information on the choice of priors and Markov chain Monte Carlo settings
☒	☐	For hierarchical and complex designs, identification of the appropriate level for tests and full reporting of outcomes
☒	☐	Estimates of effect sizes (e.g. Cohen's *d*, Pearson's *r*), indicating how they were calculated

Our web collection on statistics for biologists contains articles on many of the points above.

Software and code

Policy information about availability of computer code

Data collection	FIJI distribution of ImageJ, GraphPad Prism v7.04, BD FACS Aria Diva v8.0, NIS-Elements Advanced Research imaging software v4.20, PClamp 10 data acquisition software (Molecular Devices).
Data analysis	Trimmomatic 0.35 software (Bolger 2014), RSEM 1.2.31 (Li 2014), TOPHAT 2.1.1 and CUFFLINKS 2.2.1 (Trapnell 2012), fastQC 0.10.1 (http://www.bioinformatics.babraham.ac.uk/projects/fastqc/), FASTX 0.0.14 (http://hannonlab.cshl.edu/fastx_toolkit/download.html), RSeQC 2.6.1 (Wang 2012), and RNA-seq-QC 1.1.8 (DeLuca 2012), segmented R package, R package sigclust (Liu 2008), R package WGCNA, custom R code and count data used to generate transcriptomics related figures (https://github.com/AllenInstitute/L5_VEN), Seurat V3 (https://satijalab.org/seurat/), Uniform Manifold Approximation and Projection (UMAP) (Leland McInnes 2018), custom analysis scripts written in Igor Pro (Wavemetrics). Custom R code used for analyses can be downloaded at: https://github.com/AllenInstitute/L5_VEN.

For manuscripts utilizing custom algorithms or software that are central to the research but not yet described in published literature, software must be made available to editors/reviewers. We strongly encourage code deposition in a community repository (e.g. GitHub). See the Nature Research guidelines for submitting code & software for further information.

Data

Policy information about availability of data

All manuscripts must include a data availability statement. This statement should provide the following information, where applicable:

- Accession codes, unique identifiers, or web links for publicly available datasets
- A list of figures that have associated raw data
- A description of any restrictions on data availability

Custom R code and count data used to generate transcriptomics related figures can be downloaded from https://github.com/AllenInstitute/L5_VEN. Raw and aligned data have been registered with dbGaP (https://www.ncbi.nlm.nih.gov/projects/gap/cgi-bin/study.cgi?study_id=phs001791.v1.p1) and have been deposited in the NeMO archive (https://nemoarchive.org/) for controlled access when that feature becomes available on NeMO.

Field-specific reporting

Please select the one below that is the best fit for your research. If you are not sure, read the appropriate sections before making your selection.

- [x] Life sciences
- [ ] Behavioural & social sciences
- [ ] Ecological, evolutionary & environmental sciences

For a reference copy of the document with all sections, see nature.com/documents/nr-reporting-summary-flat.pdf

Life sciences study design

All studies must disclose on these points even when the disclosure is negative.

Sample size	Single nuclei were isolated from post-mortem brains of 2 donors. This allowed us to collect nuclei from high quality specimens that met stringent quality control metrics while also confirming that transcriptomic clusters were consistent between donors and not driven by technical artifacts. Cell counts were conducted on 3-5 donors. Electrophysiology data was collected from one donor.
Data exclusions	To remove data from low-quality samples before downstream analysis, we implemented a random forest machine-learning classification approach (Boldog 2018, Aevermann 2017).The random forest model was then applied to the data and final classifications were determined. A Pass confidence cutoff of 0.6 or greater was used to select single-nuclei data for downstream analysis. Using this random forest model applied to the entire layer 5 dataset, 78% of 1,118 single-nuclei samples passed quality control. Clusters containing cells from only a single donor as well as nuclei mapping to low-quality outlier clusters (n=318) were also excluded from further analysis.
Replication	Flow cytometry data were reproducible across human tissue specimens and across different nuclei isolations from individual tissue donors.
Randomization	All human specimens were controls and were therefore allocated into the same experimental group. Randomization was not used.
Blinding	Human specimens were de-identified and assigned a unique numerical code. Researchers had access to basic information about donors (age, sex, ethnicity) as well as the unique numerical code assigned to each donor.

Reporting for specific materials, systems and methods

We require information from authors about some types of materials, experimental systems and methods used in many studies. Here, indicate whether each material, system or method listed is relevant to your study. If you are not sure if a list item applies to your research, read the appropriate section before selecting a response.

Materials & experimental systems

n/a	Involved in the study	
[ ]	[x]	Antibodies
[x]	[ ]	Eukaryotic cell lines
[x]	[ ]	Palaeontology
[x]	[ ]	Animals and other organisms
[ ]	[x]	Human research participants
[x]	[ ]	Clinical data

Methods

n/a	Involved in the study	
[x]	[ ]	ChIP-seq
[ ]	[x]	Flow cytometry
[x]	[ ]	MRI-based neuroimaging

Antibodies

Antibodies used	Mouse monoclonal anti-NeuN antibody (clone A60, MAB377, Millipore)<br>Goat anti-mouse Alexa Fluor 594 (A-11032, ThermoFisher)
Validation	Mouse monoclonal anti-NeuN antibody - Routinely evaluated by immunohistochemistry on brain tissue.<br><br>Immunohistochemistry(paraffin) Analysis:<br>NeuN (cat. # MAB377) staining pattern/morphology in rat cerebellum. Tissue pretreated with Citrate, pH 6.0. This lot of antibody was diluted to 1:100, using IHC-Select® Detection with HRP-DAB. Immunoreactivity is seen as nuclear staining in the neurons in the granular layer. Note that there is no signal detected in the nucleus of Purkinje cells.<br>Optimal Staining With Citrate Buffer, pH 6.0, Epitope Retrieval: Rat Cerebellum<br><br>Goat anti-mouse Alexa Fluor 594 - the manufacturer provides a Certificate of Analysis that lists detailed quality control and product qualification information for each product.

Human research participants

Policy information about studies involving human research participants

Population characteristics	Single nucleus RNA-seq was conducted using tissues from two Caucasian male postmortem donors aged 50 and 54.Tissue for electrophysiology came from peri-tumor insula tissue that was removed from the brain of a 68-year-old female patient to access a deep brain tumor located in the left insula/putamen region.
Recruitment	Postmortem specimens: Males and females 18 – 68 years of age with no known history of neuropsychiatric or neurological conditions ('control' cases) were considered for inclusion in this study (Extended Data Table 1). De-identified postmortem human brain tissue was collected after obtaining permission from decedent next-of-kin. Neurosurgical specimens: recruitment of patients is performed outside of the supervision of the Allen Institute by a hospital-appointed case coordinator under the authority of the IRB of the participating hospital.
Ethics oversight	Postmortem donors: After obtaining permission from decedent next-of-kin, postmortem adult human brain tissue was collected by the San Diego Medical Examiner's office and provided to the Allen Institute for Brain Science. All tissue collection was performed in accordance with the provisions of the Uniform Anatomical Gift Act described in Health and Safety Code §§ 7150, et seq., and other applicable state and federal laws and regulations. The Western Institutional Review Board reviewed tissue collection processes and determined that they did not constitute human subjects research requiring IRB review. Neurosurgical specimens: Tissue procurement from neurosurgical donors was performed outside of the supervision of the Allen Institute at local hospitals, and tissue was provided to the Allen Institute under the authority of the IRB of each participating hospital. A hospital-appointed case coordinator obtained informed consent from donors prior to surgery.

Note that full information on the approval of the study protocol must also be provided in the manuscript.

Flow Cytometry

Plots

Confirm that:

- [x] The axis labels state the marker and fluorochrome used (e.g. CD4-FITC).
- [x] The axis scales are clearly visible. Include numbers along axes only for bottom left plot of group (a 'group' is an analysis of identical markers).
- [x] All plots are contour plots with outliers or pseudocolor plots.
- [x] A numerical value for number of cells or percentage (with statistics) is provided.

Methodology

Sample preparation	For RNA-sequencing experiments, frontoinsula (FI) was identified on slabs of interest and vibratome sectioned as described (Boldog 2018, Hodge 2018). Layer 5 was microdissected from vibratome sections stained with fluorescent Nissl. Mouse monoclonal anti-NeuN antibody (EMD Millipore, MAB377) was applied to nuclei preparations followed by secondary antibody staining (goat anti mouse Alexa Fluor 594, ThermoFisher), and single-nucleus sorting was carried out on a BD FACSAria Fusion instrument (BD Biosciences) using a 130µm nozzle following a standard gating procedure as previously described (Boldog 2018, Hodge 2018). Approximately 10% of nuclei were NeuN–negative non-neuronal nuclei. Single nuclei were sorted into 96-well PCR plates (ThermoFisher Scientific) containing 2µL of lysis buffer (0.2% Triton-X 100, 0.2% NP-40 (Sigma Aldrich), 1 U/µL RNaseOut (ThermoFisher Scientific), PCR-grade water (Ambion), and ERCC spike-in synthetic RNAs (Ambion). 96-well plates were snap frozen and stored at –80°C until use.
Instrument	BD FACS Aria Fusion
Software	BD FACS Aria Diva v8.0
Cell population abundance	We intentionally sorted ~10% NeuN-negative (non-neuronal) and ~90% NeuN-positive (neuronal) nuclei to enrich for neurons.
Gating strategy	Nuclei were first gated based on size (forward scatter area, FSC-A) and granularity (side scatter area, SSC-A). Nuclei were then gated on DAPI fluorescence, followed by gates to exclude doublets and aggregates (FSC-single cells, SSC-single cells). Lastly, nuclei were gated based on NeuN PE ignal (NeuN-PE-A) to differentiate neuronal (NeuN+) and non-neuronal (NeuN-) nuclei.

- [x] Tick this box to confirm that a figure exemplifying the gating strategy is provided in the Supplementary Information.
