# Supplementary material for: Transcriptomic evidence that von Economo neurons are regionally specialized extratelencephalic-projecting excitatory neurons
Source: Nat Commun. 2020 Mar 3;11:1172. doi: 10.1038/s41467-020-14952-3 (PMC7054400; doi:10.1038/s41467-020-14952-3)
Supplement: Supplementary file 2 — Peer Review [file 41467_2020_14952_MOESM2_ESM.pdf]

Editorial Note: This manuscript has been previously reviewed at another journal that is not operating a transparent peer review scheme. This document only contains reviewer comments and rebuttal letters for versions considered at *Nature Communications.*

REVIEWERS' COMMENTS:

Reviewer #1 (Remarks to the Author):

The authors have addressed all of my previous comments.

Reviewer #2 (Remarks to the Author):

Manuscript by Hodge et al investigates the molecular and physiological properties of a population of neurons called the von Economo neurons that are not commonly found in mouse, but have been implicated in a range of human disorders, including fronto-temporal dementia and Autism. Identifying cell types in the mammalian brain is a major challenge in the field, particularly human and non-human primate. Here, the researchers employ cutting edge technologies to identify molecular markers of von Economo neurons and leverage their recently developed slice culture models to identify their physiological properties. The study represents an important step towards understanding the biology of these cells that unlock new avenues for studying these cells. I find this paper to be comprehensive, well written, and suitable for publication in Nature communications.

The authors perform excellent an in-depth analysis of their dataset, but occasionally make extrapolations that I don’t think are suitable for results section, such as the comment in line 195 regarding the mapping of projections based on inter-species homology. Until proven, this is still a hypothesis, and therefore I would recommend relocating this sentence to the discussion, where one might find comments about likely but untested hypotheses. Developmentally transient states not measured in this study could diversify projection patterns even if the gene expression patterns are conserved between adult states.

Our ref: NCOMMS-19-33455-T
15th January 2020

REVIEWERS' COMMENTS:

Reviewer #1 (Remarks to the Author):

The authors have addressed all of my previous comments.

We appreciate the reviewer's time and are happy to hear that we have addressed all concerns.

Reviewer #2 (Remarks to the Author):

Manuscript by Hodge et al investigates the molecular and physiological properties of a population of neurons called the von Economo neurons that are not commonly found in mouse, but have been implicated in a range of human disorders, including fronto-temporal dementia and Autism. Identifying cell types in the mammalian brain is a major challenge in the field, particularly human and non-human primate. Here, the researchers employ cutting edge technologies to identify molecular markers of von Economo neurons and leverage their recently developed slice culture models to identify their physiological properties. The study represents an important step towards understanding the biology of these cells that unlock new avenues for studying these cells. I find this paper to be comprehensive, well written, and suitable for publication in Nature communications.

We appreciate the reviewer's time and are excited about the positive comments; in particular, that our study "*represents an important step towards understanding the biology of these cells that unlock new avenues for studying these cells*."

The authors perform excellent an in-depth analysis of their dataset, but occasionally make extrapolations that I don't think are suitable for results section, such as the comment in line 195 regarding the mapping of projections based on inter-species homology. Until proven, this is still a hypothesis, and therefore I would recommend relocating this sentence to the discussion, where one might find comments about likely but untested hypotheses. Developmentally transient states not measured in this study could diversify projection patterns even if the gene expression patterns are conserved between adult states.

While we feel that conserved projection mapping is quite likely, it is a fair point that we don't have any projection data in human and that this hypothesis would fit in the Discussion. Since we already discuss this topic extensively in the Discussion, we have addressed this reviewer remark by removing the sentence on line 195 from the manuscript.
